# Evidencing the clinical and economic burden of musculoskeletal disorders in Tanzania: paving the way for urgent rheumatology service development

**DOI:** 10.1093/rap/rkad110

**Published:** 2023-12-12

**Authors:** Emma Laurie, Stefan Siebert, Nateiya Yongolo, Jo E B Halliday, Sanjura M Biswaro, Stefanie J Krauth, Kajiru Gad Kilonzo, Blandina T Mmbaga, Emma McIntosh, NIHR Global Health Group, Sanjura Biswaro, Christopher Bunn, Edith Chikumbu, Jo Coast, Mia Crampin, Manuela Deidda, Eleanor Grieve, Jo Halliday, Viktor Katiti, Clive Kelly, Kajiru Kilonzo, Emma Laurie, Emma McIntosh, Blandina Theophil Mmbaga, Gloria Moshi, Elizabeth Msoka, Febronia Shirima, Stefan Siebert, Jennika Virhia, Richard Walker, Sally Wyke

**Affiliations:** School of Geographical and Earth Sciences, University of Glasgow, Glasgow, UK; School of Infection & Immunity, University of Glasgow, Glasgow, UK; Department of Clinical Research, Kilimanjaro Clinical Research Institute, Moshi, United Republic of Tanzania; Department of Internal Medicine, Kilimanjaro Clinical Medical University College, Moshi, United Republic of Tanzania; School of Biodiversity, One Health & Veterinary Medicine, University of Glasgow, Glasgow, UK; Department of Clinical Research, Kilimanjaro Clinical Research Institute, Moshi, United Republic of Tanzania; School of Health & Wellbeing, University of Glasgow, Glasgow, UK; Department for Internal Medicine, Kilimanjaro Christian Medical Centre, Moshi, United Republic of Tanzania; Department of Clinical Research, Kilimanjaro Clinical Research Institute, Moshi, United Republic of Tanzania; Department of Internal Medicine, Kilimanjaro Clinical Medical University College, Moshi, United Republic of Tanzania; School of Health & Wellbeing, University of Glasgow, Glasgow, UK

**This editorial refers to ‘Musculoskeletal (MSK) disorders with arthritis screening in Tanzania: new insights into the growing clinical, economic and societal burden of non-communicable disease’, by Mmbaga BT *et al*., https://eprints.gla.ac.uk/300074/.**

Since 1990, there has been a dramatic rise in non-communicable diseases (NCDs) within low- and middle-income countries (LMICs); the burden of NCDs in LMICs rose from accounting for 39% of disability-adjusted life years in 1990 to 66% in 2019 [1]. In response to this growing burden, global, regional and national health institutions have become increasingly active in orchestrating a response to NCDs. The global response to NCDs has prioritized cardiovascular disease, cancer, chronic respiratory disease and diabetes, with the World Health Organization Regional Office in Africa adding region-specific NCD burdens, such as sickle cell disease, to broaden priorities [2]. Amidst this galvanization of efforts to tackle NCDs, musculoskeletal (MSK) conditions have been relatively overlooked and neglected, resulting in an urgent need for service development to respond to MSK conditions in LMICs [1].

The low status of MSK conditions within the health landscape does not align with the burden of MSK conditions [1]. MSK conditions represented 17% of years lived with disability in 2019 globally and ‘account[ed] for more than 75% of disease burden for NCDs and injuries for the poorest billion people aged 5–50 years and greater than 40 years’ [1]. Furthermore, in LMICs, the prevalence of MSK conditions increased by 60% between 1990 and 2010 [3]. It is anticipated this upward trend will continue, with the future impacts of MSK disorders in LMICs set to be compounded by demographic and lifestyle changes [3]. Although regional summary health estimates can help to illustrate the broad health, economic and social impacts of MSK conditions, the lack of empirical data from LMICs might be underestimating or misrepresenting the burden [4]. Here, place-specific understanding of the impacts of MSK conditions is vital to implementation of increased and tailored resourcing to improve health workforce knowledge and training, therapy availability, treatment guidelines and appropriate, contextually informed interventions and support [1, 5].

Better place-specific understanding is crucial; although global health impacts are estimated, and biological markers of MSK are universal, MSK conditions are experienced and mediated in place and shaped by social worlds, cultural norms and the ability of health systems to treat individuals, all of which influence the understanding and impacts of MSK conditions [5, 6]. In Tanzania, the inaugural *Strategic and Action Plan for the Prevention and Control for the Prevention and Control of Non-Communicable Diseases in Tanzania* (2016–2020) laid out the need for the health service to expand its focus on communicable diseases to include NCDs; it made little mention of MSK conditions, reflective of the global policy landscape. Recent UK National Institute for Health (NIHR)-funded research, however, has shed light on the prevalence and the clinical and economic burden of MSK disorders [5, 7].

As sketched out by Kilonzo *et al.* [8], a multidisciplinary approach established critical prevalence data and economic, social and cultural impacts for those living with MSK conditions (see Fig. 1). Although retrospective analysis of hospital records reveals a huge underdiagnosis of MSK disorders within the health system [5], clinical screening of >2500 households within the Hai District of Tanzania found 1 in 17 people (5.9%) living with confirmed joint problems; 1 in 20 (5%) with degenerative ‘wear and tear’ arthritis, and 1 in 100 with inflammatory arthritis [5]. Those living with confirmed joint problems experienced significant economic impacts, incurring two to three times higher health-care costs than those without MSK conditions. For many with MSK disorders, this involved spending >10% of their income on health care, surpassing the World Health Organization’s classification of ‘catastrophic’ health-care expenditure, where their ability to meet basic needs becomes compromised [5]. Economic impacts were coupled with, and compounded by, a reduced ability to work, causing stress and worry [6]. The associated pain and restriction of joint movement reduced the ability of an individual to conduct essential self-care (from going to the toilet to cleaning themselves) or partaking in social and community events; the cumulative effect was found to cause a 25% reduction in the health-related quality of life of those with MSK conditions compared with those without [5].

**Figure 1.**
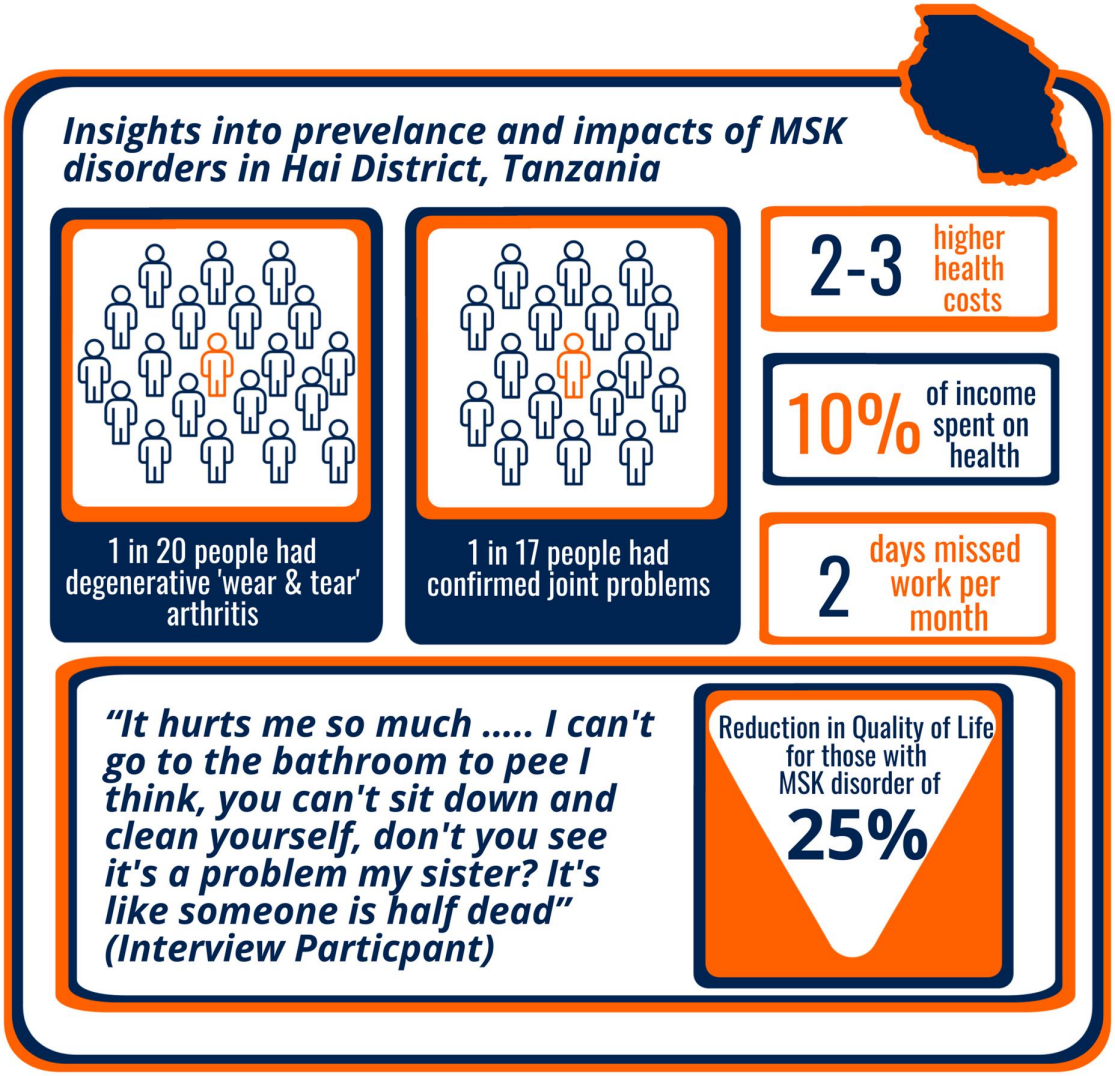
Insights into the prevalence and impact of musculoskeletal disorders in Hai District, Tanzania. After Mmbaga *et al.* (2023) [5]. MSK: musculoskeletal

Such insights reveal, for the first time, the full extent of the clinical and resulting economic burden of MSK disorders within parts of Tanzania, a burden only set to rise as demographic and lifestyle changes play out over the coming decades and which will, no doubt, be replicated throughout the country and region [3]. To respond to the growing burden, we call for ensuring MSK disorders are included in the suite of NCD challenges faced by LMICs to ensure that policies are devised to address the evidenced burdens. As detailed elsewhere, models of care for MSK disorders require multi-level responses [9]. Our findings in Tanzania reinforce this call; central to this, we recommend increased training for health workers at all levels to improve the diagnosis and management of MSK conditions. We suggest incorporating MSK diagnostic training within university curricula, continuous professional development programmes, and support for specialist training [3]. To do so effectively requires practical training resources appropriate for populations, including visual training aids that illustrate how MSK disorders display in diverse populations [10] and the development of clinical guidelines and treatment options suited to places and populations [3].

To do so requires recognition and prioritization of MSK disorders in NCD initiatives. For decades, with a rigid focus on infectious diseases, there existed a category dubbed ‘neglected tropical diseases’; we must ensure that as the health policy agenda opens up to respond to NCDs, we do not replicate a similar class of neglected NCDs, of which MSK conditions are at risk of being the foremost.

## Data Availability

Summary data are available on request.
